# First Apocarotenoids Profiling of Four Microalgae Strains

**DOI:** 10.3390/antiox8070209

**Published:** 2019-07-06

**Authors:** Mariosimone Zoccali, Daniele Giuffrida, Fabio Salafia, Carmen Socaciu, Kari Skjånes, Paola Dugo, Luigi Mondello

**Affiliations:** 1 Department of Chemical, Biological, Pharmaceutical and Environmental Sciences, University of Messina, 98166 Messina, Italy; 2 Department of Biomedical, Dental, Morphological and Functional Imaging Sciences, University of Messina, 98125 Messina, Italy; 3 PROPLANTA-Research Centre for Applied Biotechnology, str. Trifoiului 12G, 400478 Cluj-Napoca, Romania; 4 Division of Biotechnology and Plant Health, The Norwegian Institute of Bioeconomy Research (NIBIO), PO115, N-1431 Ås, Norway; 5 Chromaleont s.r.l., c/o Department of Chemical, Biological, Pharmaceutical and Environmental Sciences, University of Messina, 98166 Messina, Italy; 6 BeSep s.r.l., c/o Department of Chemical, Biological, Pharmaceutical and Environmental Sciences, University of Messina, 98166 Messina, Italy; 7 Unit of Food Science and Nutrition, Department of Medicine, University Campus Bio-Medico of Rome, 00128 Rome, Italy

**Keywords:** carotenoid derivatives, microphytes, supercritical fluid extraction-supercritical fluid chromatography-tandem mass spectrometry, hyphenated techniques

## Abstract

Both enzymatic or oxidative carotenoids cleavages can often occur in nature and produce a wide range of bioactive apocarotenoids. Considering that no detailed information is available in the literature regarding the occurrence of apocarotenoids in microalgae species, the aim of this study was to study the extraction and characterization of apocarotenoids in four different microalgae strains: *Chlamydomonas* sp. CCMP 2294, *Tetraselmis chuii* SAG 8-6, *Nannochloropsis gaditana* CCMP 526, and *Chlorella sorokiniana* NIVA-CHL 176. This was done for the first time using an online method coupling supercritical fluid extraction and supercritical fluid chromatography tandem mass spectrometry. A total of 29 different apocarotenoids, including various apocarotenoid fatty acid esters, were detected: apo-12’-zeaxanthinal, β-apo-12’-carotenal, apo-12-luteinal, and apo-12’-violaxanthal. These were detected in all the investigated strains together with the two apocarotenoid esters, apo-10’-zeaxanthinal-C4:0 and apo-8’-zeaxanthinal-C8:0. The overall extraction and detection time for the apocarotenoids was less than 10 min, including apocarotenoids esters, with an overall analysis time of less than 20 min. Moreover, preliminary quantitative data showed that the β-apo-8’-carotenal content was around 0.8% and 2.4% of the parent carotenoid, in the *C. sorokiniana* and *T. chuii* strains, respectively. This methodology could be applied as a selective and efficient method for the apocarotenoids detection.

## 1. Introduction

**Figure 1:**
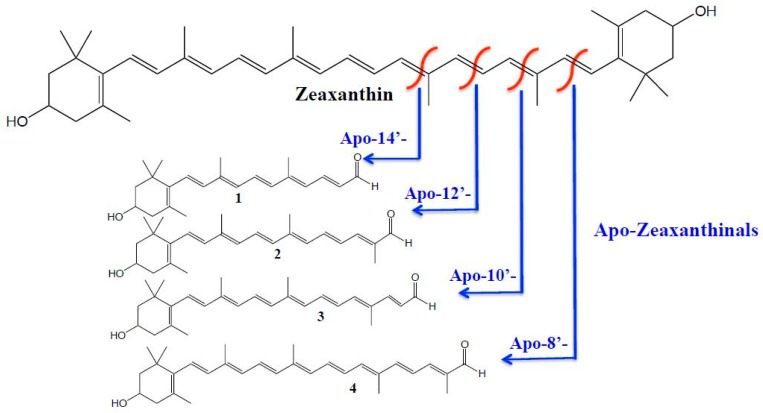
Zeaxanthin oxidative cleavages sites producing various apozeaxanthinals; **1**. Apo-14’-Zeaxanthinal; **2**. Apo-12’-Zeaxanthinal; **3**. Apo-10’-Zeaxanthinal; **4**. Apo-8’-Zeaxanthinal. Reprint with permission from [14].

The carotenoids composition of microalgae has been widely investigated [1,2,3,4] and, recently, the occurrence of carotenoids esters in microalgae has also been reported [5]. The carotenoid profiles are known to vary greatly between species, as are the algae’s ability to accumulate different carotenoids during stress exposure [6]. The production of carotenoids from microalgae is continuously growing since natural and controlled production sources of carotenoids are highly desirable because of their economic and environmental positive aspects [7]. Carotenoids are tetraterpenoidic lipophilic compounds with health beneficial properties, such as antioxidant activity [8,9], composed of two main classes: the carotenes that are hydrocarbons molecules and the xanthophylls that are oxygenated ones. It is very common in nature to find xanthophylls esterified with fatty acids; in fact, xanthophyll esters have greater stability then free xanthophylls. Different analytical methods for extraction and analysis of carotenoids in microalgae samples were reported mainly based on liquid extraction and liquid chromatography approaches [10], but they were also based on supercritical fluids approaches [11,12]. Both enzymatic or oxidative carotenoids cleavages often occur in plants that produce a wide range of bioactive apocarotenoids [13,14]. Possible zeaxanthin oxidative cleavage sites that produce various apozeaxanthinals are shown in Figure 1. There is a growing interest in the investigation of apocarotenoids in food, food products, and mammals due to the beneficial effects attributed to them [14,15,16]. Very recently, Zoccali et al. [17] and Giuffrida et al. [18] reported on the first application of a supercritical fluid extraction-supercritical fluid chromatography-mass spectrometry (SFE-SFC-MS) methodology for, respectively, the carotenoids and the apocarotenoids determination in different food matrices. To the best of the authors knowledge, no detailed data is available in the literature on the apocarotenoids occurrence in microalgae. Therefore, the aim of this investigation was to determine the occurrence of apocarotenoids in four selected different microalgae strains: *Chlamydomonas* sp CCMP 2294, *Tetraselmis chuii* SAG 8-6, *Nannochloropsis gaditana* CCMP 526, and *Chlorella sorokiniana* NIVA-CHL 176.

## 2. Materials and Methods

### 2.1. Chemicals

All chemicals were obtained from Merck Life Science (Merck KGaA, Darmstadt, Germany). A series of β-apocarotenals, apozeaxanthinals, and ε-apoluteinals were generated by oxidative cleavages of the parent carotenoids as reported in references [19,20,21]; moreover, the β-apo-8’-Carotenal standard was purchased from CaroteNature GmbH (Münsingen, Switzerland). The standards of the parent carotenoids, namely, β-carotene, zeaxanthin, and lutein were obtained from Extrasynthese (Genay, France). 

### 2.2. Strain Selection and Biomass Production

The following four different microalgae strains were acquired from culture collections and were thusly investigated: 

*Chlamydomonas* sp CCMP 2294 was obtained from Bigelow Laboratory for Ocean Sciences (NCMA), USA.

Artic marine collection site: Baffin Bay, between Ellesmere Island, Canada and Greenland (77.8136° N 76.3697° W, sea ice core), belonging to the Chlamydomonadaceae family.

Cultivated under the following conditions: Light intensity: 70–80 µmol/m^2^/s, temperature 4 °C, 6 L cultures in 10 L flasks bubbled with air added 1% CO_2_, growth medium L1 [22].

*Tetraselmis chuii* SAG 8-6 was obtained from SAG Culture Collection of Algae, Germany.

Temperate marine collection site: Scotland, Millport, Clyde estuary (55.751383/–4.931953, 600 m), belonging to the Chlorodendraceae family.

Cultivated under the following conditions: Light intensity: 130 µmol/m^2^/s, temperature 25 °C, 1 L cultures in 2 L Erlenmeyer flasks on shaking table, air with 3% CO_2_ was added to headspace; Light intensity: 50 µmol/m^2^/s, temperature 22 °C, 5–6 L cultures in 10 L flasks bubbled with air added 3% CO_2_, both with growth medium L1.

*Nannochloropsis gaditana* CCMP 526 (recently renamed after full genome completion, as *Microchloropsis gaditana*) was obtained from Bigelow Laboratory for Ocean Sciences (NCMA), USA.

Temperate marine collection site: Morocco, Lagune de Oualidia, (32.8333° N 9° W), belonging to the Eustigmataceae family.

Cultivated under the following conditions: Light intensity: 130 µmol/m^2^/s, temperature 25 °C, 1 L cultures in 2 L Erlenmeyer flasks on shaking table, air with 3% CO_2_ was added to headspace; Light intensity: 50 µmol/m^2^/s, temperature 22 °C, 5–6L cultures in 10 L flasks bubbled with air added 3% CO_2_, both with growth medium L1.

*Chlorella sorokiniana* NIVA-CHL 176 was obtained from The Norwegian Culture Collection of Algae (NORCCA), Norway.

Temperate fresh water collection site: Waller Creek, University of Texas, Austin, USA, belonging to the Chlorellaceae family.

Cultivated under the following conditions: Light intensity: 150 µmol/m^2^/s, temperature 25 °C, 1 L cultures in 1,2 L flat flasks bubbled with air added 2–3% CO_2_. Growth medium Tris-Acetate-Phosphate (TAP) [23], modified by replacing acetate with HCl. 

All the above described microalgae biomasses were lyophilized before apocarotenoids analyses.

### 2.3. Sample Preparation

The microalgae samples (1 mg) were placed in the extraction vessel in the SFE unit. A 0.2 mL extraction vessel was used. Supercritical CO_2_ and CH_3_OH were then utilized to perform the extraction and then the chromatography as reported in Section 2.5.

### 2.4. SFE-SFC-MS Instrumentation

The SFE-SFC-MS analyses were carried out on a Shimadzu Nexera-UC system (Shimadzu, Kyoto, Japan), composed of a CBM-20A controller, an SFE-30A module for supercritical fluid extraction, two LC-20AD_XR_ dual-plunger parallel-flow pumps, an LC-30AD_SF_ CO_2_ pump, two SFC-30A back pressure regulator, a DGU degasser, a CTO-20AC column oven, a SIL-30AC autosampler, an LCMS-8050 mass spectrometer equipped with an atmospheric pressure chemical ionization (APCI) source. The all system was controlled by the LabSolution ver. 5.8 (Shimadzu, Kyoto, Japan).

### 2.5. SFE-SFC-MS Analytical Conditions

**Figure 2:**
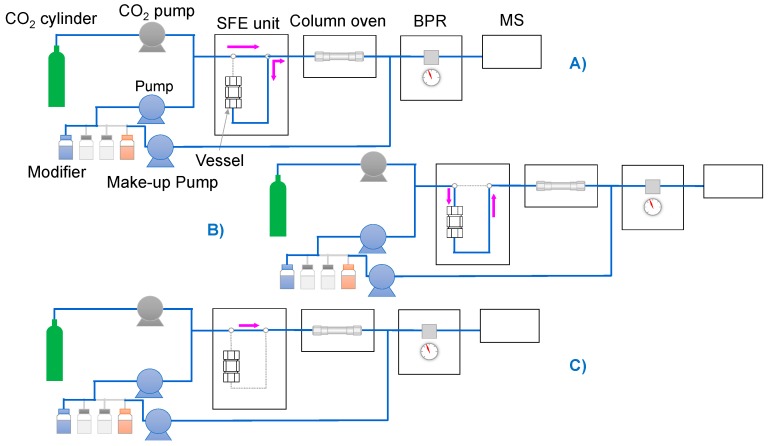
Scheme of the supercritical fluid extraction-supercritical fluid chromatography-mass spectrometry (SFE-SFC-MS) system: (**A**) Static extraction mode, (**B**) Dynamic extraction mode, (**C**) Analysis mode. Reprinted with permission from [17].

A scheme of the SFE-SFC-MS system is reported in Figure 2 and described in detail in Zoccali et al. [17]. The system operates in three different steps: (1) SFE static extraction mode, (2) SFE dynamic extraction mode, and (3) SFC analysis. During the static extraction mode, the vessel was pressurized for 3 min (Figure 2A), then the extraction was carried out in the dynamic mode for one min (Figure 2B). During this step, the mobile phase flows through the vessel continuously and the extracts are transferred into the analytical column. After the SFEs steps 1 and 2, the analytes undergo the SFC analysis (Figure 2C).

The SFE conditions were as follows: 0–3 min static extraction mode, and 3–4 min dynamic extraction mode; Extraction vessel temperature: 80 °C. Back pressure regulator: 150 bar. 

Solvent (A) CO_2_ and solvent (B) CH_3_OH; Gradient: From 0 to 3 min, 5% of B; then from 3 to 4 min, 10% of B. Flow rate: 2 mL/min.

The SFC conditions were as follows: Solvent (A) CO_2_ and solvent (B) CH_3_OH. Gradient: from 4 to 6.0 min 0% B, from 6 to 14 min increasing from 0 to 40% in 8 min, then 40% for 5 min. Flow rate: 2 mL/min.

Separation were carried out on an Ascentis Express C30, 150 mm × 4.6 mm × 2.7 μm *_d.p_*. Merck Life Science (Merck KGaA, Darmstadt, Germany. The used eluents were: A, CO_2_; B CH_3_OH; make-up solvent, CH_3_OH; 35 °C was the column oven temperature and 150 bar was the regulator back pressure. The injection volume for standards was 3 µL. The MS was set as follows: Acquisition mode: SCAN in negative mode (−) and selected ion monitoring (SIM) (−). Interface temperature: 350 °C; DL temperature: 200 °C; block heater temperature: 200 °C; nebulizing gas flow (N_2_) 3 L/min; drying gas flow (N_2_) 5 L/min; Full scan range: 200–1200 *m*/*z*; event time: 0.05 sec for each event. The available standards, full scan, SIM, and multiple reaction monitoring (MRM) experiments were used for the apocarotenoid identifications. Transitions in the MS/MS experiments were previously optimized for the β-apocarotenals and apozeaxanthinals by Giuffrida et al. [20] and for ε-apoluteinals by Zoccali et al. [21]. β-Carotene and β-apo-8’-carotenal were quantitatively determined by multiple extractions as reported in Zoccali et al. [17]. Six-point calibration curves were constructed in the 0.1–20 mg L^−1^ range. The derived calibration curves had a coefficient of determination (R^2^) of 0.9996 and 0.9991, respectively, for β-carotene and β-apo-8’-carotenal. Linearity was further confirmed using Mandel’s fitting test. Limits of detection (LoD) were 0.03 and 0.04 mg L^−1^, while limits of quantification (LoQ) were 0.091, 0.134 mg L^−1^, respectively, for β-carotene and β -apo-8’-carotenal. Further, they were calculated by multiplying the standard deviation of the standard area at the lowest concentration level, three and ten times, respectively, and then were divided by the slope of the calibration curve.

## 3. Results and Discussion

Microalgae represents one of the most promising sources of bioactive molecules, including carotenoids [24,25]. In fact, they have the ability to adapt and grow in many different environmental conditions, going from tropic to temperate and artic waters [26]. In addition, many algae strains representing most habitats have stress handling mechanisms that frequently involve increased carotenoid production when exposed to unfavorable environmental conditions [27,28]. The actual knowledge of the carotenoids biosynthetic trails on microalgae is still mainly coming from plant studies [25]. 

The carotenoids composition of the selected four different microalgae species belonging to different botanical families and having different geographical origin—*Chlamydomonas* sp., *T. chuii*, *N. gaditana*, and *C. sorokiniana*—were reported in [29,30,31,32,33,34], although the selected psychrophilic *Chlamydomonas* sp. strain has not been previously explored. Interestingly, the possible occurrence of apocarotenoids in those microalgae species had never been investigated before. Extremophile species—in this case, the psychrophilic one—have mechanisms for tolerating conditions that would quickly kill other strains and probably have secondary metabolites not present in temperate species [35]. Some *Chlamydomonas* spp. and strains of *C. sorokiniana* have been reported to produce lutein as the main carotenoid [31,33]. *T. chuii* is a food approved species and has been reported to accumulate α and β-carotenes, whereas *N. gaditana*, which is frequently used in aquaculture feed due to its high eicosapentaenoic fatty acid (EPA) content, has been reported to accumulate violaxanthin and zeaxanthin [30,34]. 

The here reported methodology allowed for the determination of the native apocarotenoids prolife in four different microalgae species for the first time; a total of 29 different apocarotenoids, including various apocarotenoid fatty acid esters, were detected. The overall extraction and detection time for all the apocarotenoids was less than 10 min, including apocarotenoids esters, with an overall analysis time less than 20 min.

Table 1 shows the overall apocarotenoids detected by SFE-SFC-APCI(+/−)/QqQ MS analysis in the four microalgae strains. SIM detections and MRM transitions were applied to all the detected apocarotenoids except for the apo-violaxanthinals and apo-fucoxanthinals that were identified only using SIM detections, due to the lack of the respective standards. 

**Figure 3:**
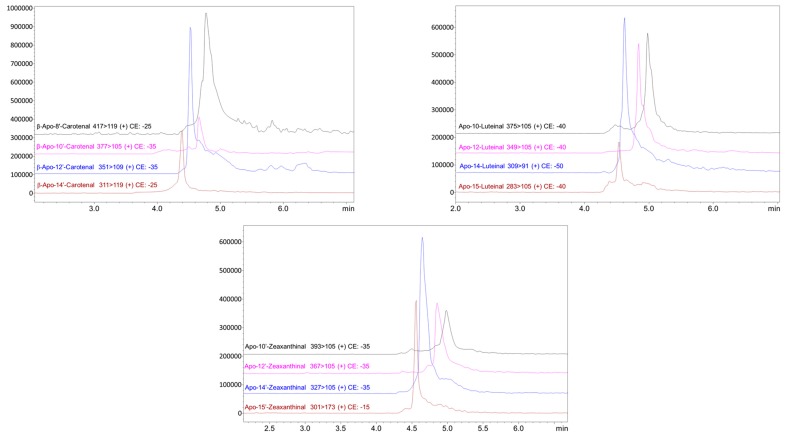
MRM analysis enlargements (transitions in APCI positive) relative to the detected β-apo-carotenals, apo-zeaxanthinals, and ε-apo-luteinals in the different microalgae strains.

Table 2 shows the overall apocarotenoids occurrence in the four microalgae strains. In general, it can be observed that the apocarotenoids were occurring in the microalgae strains in a scattered order although apo-12’-zeaxanthinal, β-apo-12’-carotenal, apo-12-luteinal, and apo-12’-violaxanthal were detected in all the investigated strains together with the two apocarotenoid esters, apo-10’-zeaxanthinal-C4:0, and apo-8’-zeaxanthinal-C8:0. The *Chlamydomonas* sp. strain showed the highest apocarotenoids occurrence among the investigated strains. In fact, 25 apocarotenoids were detected in this microalga. As far as we know this is the first detailed study on the apocarotenoids occurrence in any microalgae species. The presence of β-apo-8’-carotenal, β-apo-10’-carotenal, and apo-12’-violaxanthal were only previously reported by Sommella et al. [36] in Spirulina supplements. In Figure 3 are shown as example, the MRM analysis enlargements (transitions in APCI positive) relative to the detected β-apo-carotenals, apo-zeaxanthinals, and ε-apo-luteinals in the different microalgae strains. Further, it can be appreciated that all the different apocarotenoids were identified in less than 6 min of SFE-SFC-MS analysis. Although the purpose of this investigation was a qualitative apocarotenoids that profiled the four different microalgae strains, the available standards allowed us to also carry out a quantitative evaluation of the β-carotene and β-apo-8’-carotenal contents in the investigated samples. The amount of β-carotene was 89.7, 46.9, 20.6, and 4.2 ng mg^−1^ respectively in the *C. sorokiniana*, *N. gaditana*, *T. chuii*, and *Chlamydomonas* sp. samples, while β-apo-8’-carotenal was detected only in *C. sorokiniana* and *T. chuii* samples, with an amount of 0.7 and 0.5 ng mg^−1^, respectively. Therefore, interestingly, considering the reported preliminary quantitative data the β-apo-8’-carotenal content was around the 0.8% and the 2.4% of the parent carotenoid in *C. sorokiniana* and *T. chuii*, respectively.

## 4. Conclusions

The SFE-SFC-MS methodology applied in this work provided the first detailed report on the apocarotenoids detection and occurrence in four microalgae strains. The applied methodology was selective and efficient for the apocarotenoids detection. The reported determination of apocarotenoids in the microalgae further demonstrates the natural occurrence of these metabolites in the natural matrices, which certainly deserve further investigation. Moreover, the detection of fatty acids esterified apocarotenoids further demonstrate the wide occurrence and importance of the esterification process in carotenoids and carotenoid derivatives [37]. The possible exploitation of microalgae also containing biologically active apocarotenoids as functional food ingredients should be further explored by the food and feed industry.

## Figures and Tables

**Table 1:** Selected ion monitoring (SIM) *m*/*z*, Multiple reaction monitoring (MRM) with quantifier (Q) and qualifier (q) transitions (Collision Energy V) and Q/q % ratio of the detected apocarotenoids in the four microalgae strains.

Apocarotenoids	SIM (−)	MRM Transition (CE)		
	*m*/*z*	Quantifier	Qualifier	Q/q %
β-Apo-8’-Carotenal	416	+ 417>119 (−25)	+ 417>105 (−35)	73
β-Apo-10’-Carotenal	376	+ 377>105 (−35)	+ 377>119 (−30)	79
β-Apo-12’-Carotenal	350	+ 351>105 (−35)	+ 351>119 (−25)	74
β-Apo-14’-Carotenal	310	+ 311>105 (−25)	+ 311>119 (−25)	77
Apo-8’-Zeaxanthinal	432	+ 433>119 (−30)	+ 433>105 (−35)	95
Apo-10’-Zeaxanthinal	392	+ 393>105 (−35)	+ 393>119 (−25)	92
Apo-12’-Zeaxanthinal	366	+ 367>105 (−35)	+ 367>119 (−30)	80
Apo-14’-Zeaxanthinal	326	+ 327>105 (−35)	+ 327>119 (−30)	61
Apo-15-Zeaxanthinal	300	+ 301>173 (−15)	+ 301>105 (−30)	57
Apo-8-Luteinal	432	+ 415>119 (−40)	+ 415>91 (−50)	95
Apo-10-Luteinal	392	+ 375>105 (−40)	+ 375>91 (−50)	91
Apo-12-Luteinal	366	+ 349>105 (−40)	+ 349>91 (−50)	90
Apo-14-Luteinal	326	+ 309>91 (−50)	+ 309>105 (−40)	55
Apo-15-Luteinal	300	+ 283>105 (−40)	+ 283>91 (−50)	95
Apo-8’-violaxanthin	448	n.d.	n.d.	
Apo-12’-violaxanthal	382	n.d.	n.d.	
Apo-14’-violaxanthal	342	n.d.	n.d.	
Apo-15’-violaxanthal	316	n.d.	n.d.	
Apo-8’-Fucoxanthinal	464	n.d.	n.d.	
Apo-10’-Fucoxanthinal	424	n.d.	n.d.	
Apo-14’-Fucoxanthinal	358	n.d.	n.d.	
Apo-15’-Fucoxanthinal	332	n.d.	n.d.	
Apocarotenoids-Esters	SIM (−)	MRM transition (CE)		
Apo-10’-Zeaxanthinal-C4:0	462	+ 463>105 (−40)	+ 463>119 (−35)	71
Apo-10’-Zeaxanthinal-C10:0	546	+ 547>105 (−35)	+ 547>119 (−30)	87
Apo-10’-Zeaxanthinal-C12:0	574	+ 575>105 (−35)	+ 575>119 (−30)	75
Apo-10’-Zeaxanthinal-C14:0	602	+ 603>105 (−40)	+ 603>119 (−30)	77
Apo-8’-Zeaxanthinal-C8:0	558	+ 559>105 (−40)	+ 559>119 (−40)	70
Apo-8’-Zeaxanthinal-C10:0	586	+ 587>119 (−40)	+ 587>105 (−40)	81
Apo-8’-Zeaxanthinal-C12:0	614	+ 615>105 (−40)	+ 615>119 (−40)	79

n.d. = not determined.

**Table 2:** Overall apocarotenoids occurrence in four microalgae strains.

Compound	Chlorella sorokiana NIVA-CHL 176	Nanochloropsis gaditana CCMP 526	Tetraselmis chui SAG 8-6	Chlamydomonas sp. CCMP 2294
Apo-8’-Zeaxanthinal	-	×	-	×
Apo-10’-Zeaxanthinal	×	-	-	×
Apo-12’-Zeaxanthinal	×	×	×	×
Apo-14’-Zeaxanthinal	×	-	×	×
Apo-15’-Zeaxanthinal	-	×	×	×
β-Apo-8’-Carotenal	×	-	×	-
β-Apo-10’-Carotenal	×	×	-	×
β-Apo-12’-Carotenal	×	×	×	×
β-Apo-14’-Carotenal	×	-	×	×
Apo-10’-Zeaxanthinal -C4:0	×	×	×	×
Apo-10’-Zeaxanthinal -C10:0	×	×	-	×
Apo-10’-Zeaxanthinal -C12:0	×	-	-	×
Apo-10’-Zeaxanthinal -C14:0	×	-	×	×
Apo-8’-Zeaxanthinal-C8:0	×	×	×	×
Apo-8’-Zeaxanthinal-C10:0	×	×	-	×
Apo-8’-Zeaxanthinal-C12:0	×	×	-	×
Apo-8-Luteinal	-	×	×	-
Apo-10-Luteinal	×	×	×	×
Apo-12-Luteinal	×	×	×	×
Apo-14-Luteinal	×	-	×	×
Apo-15-Luteina	×	-	-	×
Apo-8’-Violaxanthin	×	-	×	×
Apo-12’-Violaxanthal	×	×	×	×
Apo-14’-Violaxanthal	×	-	×	×
Apo-15’-Violaxanthal	-	×	×	×
Apo-8’-Fucoxanthinal	-	×	×	×
Apo-10’-Fucoxanthinal	-	×	-	-
Apo-14’-Fucoxanthinal	×	-	-	-
Apo-15’-Fucoxanthinal	×	-	×	×

× = Detected; - = not detected.
